# Automated Identification of Accessory Mental Foramen Using Cone-Beam Computed Tomography and Convolutional Neural Networks

**DOI:** 10.1016/j.identj.2026.109428

**Published:** 2026-02-24

**Authors:** Zuhal Ovuz, Eda Gursu Sahin, Taha Etem

**Affiliations:** a Faculty of Dentistry, Department of Maxillofacial Radiology, Cankiri Karatekin University, Cankiri, Türkiye; b Faculty of Dentistry, Department of Endodontics, Cankiri Karatekin University, Cankiri, Türkiye; c Faculty of Engineering, Department of Computer Engineering, Cankiri Karatekin University, Cankiri, Türkiye

**Keywords:** CBCT, Deep learning, Accessory mental foramen, Convolutional neural network, Artificial intelligence

## Abstract

**Introduction and aims:**

To develop and evaluate a deep learning-based system for automatic detection of the accessory mental foramen (AMF) using cone-beam computed tomography (CBCT) images, and to compare the detection accuracy and clinical reliability performance of two convolutional neural network (CNN) architectures for this model.

**Methods:**

A total of 3000 CBCT scans were retrospectively screened. After expert evaluation, 700 CBCT scans exhibiting AMFs were identified. For comparative analysis, 700 CBCT scans with normal mental-foramen anatomy were selected as the matched control group. A custom lightweight CNN and a ResNet-50 model were trained for binary classification of AMF presence. Model performance was evaluated by determining accuracy, precision, recall, and the F1-score. Gradient-weighted class activation mapping (Grad-CAM) visualisation was employed to assess the anatomical relevance of the models’ attention maps. Statistical analyses were performed to compare the diagnostic performance of the two networks.

**Results:**

The ResNet-50 model achieved superior performance (overall accuracy: 85.8% for ResNet-50 vs 71.1% for the custom CNN). With the ResNet-50 model, anomaly recall improved from 0.68 to 0.88, reducing missed detections by 63%. Grad-CAM analysis demonstrated that the models focused primarily on anatomically valid regions around the MF, confirming the interpretability and clinical relevance of the models.

**Conclusions:**

Automatic detection of the AMF using CBCT and deep learning represents a reliable, objective, and efficient diagnostic approach that minimises observer bias and enhances clinical decision-making.

**Clinical relevance:**

Deep learning-based detection of AMFs on CBCT can enhance diagnostic accuracy and reduce the risk of surgical complications by providing consistent, observer-independent evaluations.

## Introduction

The inferior alveolar nerve, artery, and vein are located in the mandibular canal (MC). During surgical, endodontic, and periodontal operations, to ensure successful nerve blockade without harming the neurovascular bundles, confirming the MC’s path and changes is essential.^1^, ^2^, ^3^ The inferior alveolar nerve typically splits into two branches in the premolar region. The incisive nerve enters the incisive teeth, whereas the mental nerve and its associated circulatory systems exit the bone through the mental foramen (MF), an anatomical feature. The mental nerve provides sensory innervation for the skin of the mental region, the buccal vestibule, the gingiva mesial to the mandibular first tooth, and the skin and mucosa of the lower lip.^4^, ^5^, ^6^ The MF is located in the anterolateral aspect of the mandible, 13 to 15 mm above the inferior border of the mandibular body. In a posterior configuration, the MF’s entrance faces upward and outward.^7^ In humans, the MF is typically solitary; additional foramina are referred to as accessory mental foramina (AMFs). The occurrence of AMFs has been reported to range from 1.4% to 10%, making it an extremely uncommon finding. Although the MF’s placement can vary greatly, it is usually found beneath the second premolar’s root or between the apices of the lower first and second premolars’ roots. However, it may appear as far posterior as the first molar or as far anterior as the canine.^8^ Even among people of the same age, sex, or ethnicity, the MF’s stance can differ substantially. Furthermore, the MF may be positioned differently on each side of the mandible in the same individual.

Typically, the mandible has one MF on each side. Anatomically, the area around the MF contains other auxiliary foramina that are smaller in diameter than the main foramen. However, the term AMF is used to describe an extra foramen that is anatomically related to the MC. Conversely, ‘nutritional foramina’ are foramina that are not directly connected to the MC.^6^^,^^9^^,^^10^ The rapid evolution of artificial intelligence (AI) is significantly reshaping clinical workflows in dentistry, from diagnostic imaging to personalized treatment planning.^11^ The AMF is as important as the MF since the neuronal and vascular branches pass through it. Understanding the location, shape, and dimensions of the MF and AMF is particularly important for surgical procedures involving this area, as well as those requiring local anaesthesia, such as genioplasty, root resection of mandibular premolars, mandibular rehabilitation after trauma, apical curettage, and dental implant placement.^12^

Several techniques have been explored to accurately locate the MF, including visual observation during surgery, cadaver dissection, periapical and panoramic radiography, computed tomography (CT), cone-beam CT (CBCT), and magnetic resonance imaging. CBCT scans employ a different acquisition method from multidetector CT (MDCT) scans. CBCT creates a cone-shaped X-ray beam that allows image acquisition in a single shot as opposed to the MDCT approach of obtaining an image as discrete slices. Using CBCT, several reconstructed pictures (such as images in axial, coronal, and sagittal views) that resemble conventional MDCT images can be obtained by reformatting the resulting volume.^9^ CBCT provides detailed information regarding the structures of the maxillofacial complex and is a useful method for detecting and evaluating anatomical differences.^10^ Additionally, three-dimensional (3D) measurements can be made using CBCT images and specialised software. Some reports have shown that 3D measurements taken from CBCT scans yield results that are fairly comparable to measurements taken from the actual skull.^11^

AI-based image analysis methods are being increasingly integrated into dentomaxillofacial radiology, particularly for automated evaluation of CBCT images. In dental imaging, deep learning approaches have been employed for tasks such as anatomical landmark detection, localisation of neural canals and foramina, and identification of clinically relevant anatomical variations. These applications can support clinicians by improving diagnostic consistency, reducing observer-dependent variability, and enhancing the accuracy of treatment planning.^12^Among the existing deep learning models, convolutional neural networks (CNNs) have demonstrated strong performance in analysing CBCT images due to their ability to learn hierarchical spatial features directly from 3D data. CBCT-based studies have shown that CNNs can reliably detect and localise maxillofacial anatomical structures, indicating their potential role as adjunctive tools supporting clinical decision-making rather than replacing expert interpretation.^13^

Building on these advancements, the objective of the present study was to develop a CNN-based model that could accurately detect the presence and anatomical location of AMFs on CBCT images. To this end, two different CNN architectures – a custom lightweight network and a ResNet-50 model – were trained and evaluated using a large, expert-annotated CBCT dataset. Subsequently, model performance was assessed using standard classification metrics, and gradient-weighted class activation mapping (Grad-CAM) was employed to verify that the networks focused on anatomically relevant regions. The results demonstrated the feasibility and reliability of AI-assisted CBCT analysis for the detection of AMFs and highlighted its potential value in enhancing diagnostic accuracy and clinical decision-making in maxillofacial radiology.^14^

## Materials and methods

Ethical approval for this study was obtained from the Non-Interventional Clinical Research Ethics Committee of Cankiri Karatekin University (Approval Code: 9e480299f39d4d9b; Approval Date: 4 December 2024). The study was conducted in full accordance with the ethical standards of the institutional and/or national research committee and with the 1964 Declaration of Helsinki and its later amendments or comparable ethical standards.

In this retrospective study, we included patients who visited the Department of Oral and Maxillofacial Radiology, Faculty of Dentistry, Cankiri Karatekin University, to undergo CBCT imaging examinations between October 2023 and May 2024. Written informed consent was obtained from all participants before inclusion in the study. All tomographic procedures were performed in accordance with standard infection control and radiographic safety protocols, including the use of lead aprons.

At the initial stage of the study, 3000 CBCT scans were retrospectively screened from the institutional archive. To ensure diagnostic reliability, this screening was independently performed by a dentomaxillofacial radiologist and an endodontist. From this dataset, 700 CBCT scans demonstrating the presence of an AMF and meeting the image quality and inclusion criteria listed below were identified. For comparison, 700 age- and sex-matched CBCT scans with normal MF anatomy were selected as the control group. Consequently, the final statistical analysis was conducted on a total of 1400 CBCT scans. To assess annotation reliability, an initial subset of 20 CBCT scans was independently evaluated by a maxillofacial radiologist and an endodontist. Both observers were blinded to each other’s assessments and to the clinical information. Interobserver agreement was quantified using Cohen’s kappa coefficient, which demonstrated high reliability (*κ* = 0.88). After this calibration step, the examiners annotated the remaining dataset, and consensus labelling was applied only in cases showing disagreement. The radiographic criteria used to distinguish AMFs from nutrient foramina included continuity with the mental canal, cortical borders, and consistent visualisation across the axial, coronal, and sagittal planes.

CBCT scans were included if they met the following criteria:

(1) covered the mandibular premolar region bilaterally,
(2) had sufficient image quality for reliable visualisation of the MF region, and
(3) were obtained from patients without a history of mandibular surgery or pathology affecting the MF area.

CBCT scans were excluded if they met any of the following criteria:

(1) severe motion artefacts or low spatial resolution,
(2) metallic artefacts obscuring the MF region,
(3) previous mandibular trauma, surgical intervention, or extensive pathological lesions in the area of interest, or
(4) incomplete anatomical coverage of the region under evaluation.

The CBCT scans were obtained using the Castellini X-Rad1 US TR10 Plus device with the following parameters: tube voltage, 90 kVp; tube current, 5 mA; and exposure time, 7 pulses/s. The voxel size and field of view were set at 0.3 mm and 10 × 8 mm, respectively. The images were evaluated under appropriate lighting conditions using a Lenovo 15.0 510 80 SR monitor.

**Fig. 1:**
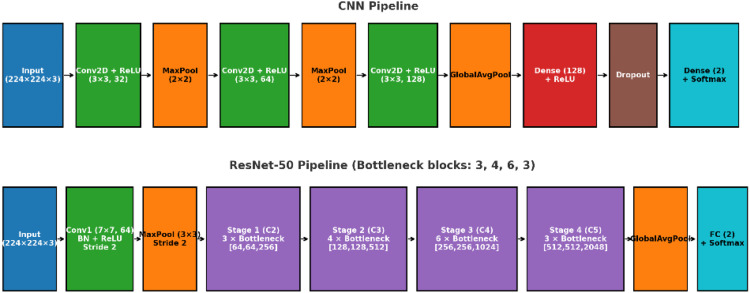
Schematic of the custom CNN (top) and ResNet-50 (bottom) pipelines used for mental-foramen detection.

Two CNN architectures were implemented for binary MF detection: a lightweight custom CNN and a ResNet-50 backbone configured with bottleneck blocks. The full model diagrams are provided in Figure 1.

Panoramic radiographs were resized to 224 × 224 pixels with three channels to match the input shape of both networks (224 × 224 × 3). Pixel intensities were standardised per image. Labels were encoded as two classes, and model outputs were interpreted as class posterior probabilities using a SoftMax layer.

### Custom CNN architecture

The custom network focused on simplicity and low parameter count.^15^ The stack for this network was as follows:

1. Conv block 1: 2-D convolution (3 × 33\times33 × 3, 32 filters) followed by rectified linear unit (ReLU).
2. Downsampling 1: 2 × 22\times22 × 2 max-pooling.
3. Conv block 2: 2-D convolution (3 × 33\times33 × 3, 64 filters) with ReLU.
4. Downsampling 2: 2 × 22\times22 × 2 max-pooling.
5. Conv block 3: 2-D convolution (3 × 33\times33 × 3, 128 filters) with ReLU.
6. Spatial aggregation: Global average pooling to convert feature maps into a 128-D vector.
7. Classifier head: Dense (128 units, ReLU), dropout for regularisation, and a final Dense layer (2 units) with SoftMax.

This design uses only 3 × 33 kernels, relies on max-pooling for spatial downsampling, and applies global average pooling to limit parameters before the classifier.

### ResNet-50 architecture

A residual network with the canonical 50-layer configuration was used.^16^ The stem contained a 7 × 77 convolution (64 filters, stride 2) with batch normalisation and ReLU, followed by 3 × 33 max-pooling (stride 2). Four residual stages with bottleneck blocks then operated at progressively reduced spatial resolutions:

• Stage 1 (C2): three bottleneck blocks with channel pattern (64, 64, 256).
• Stage 2 (C3): four bottleneck blocks with (128, 128, 512).
• Stage 3 (C4): six bottleneck blocks with (256, 256, 1024).
• Stage 4 (C5): three bottleneck blocks with (512, 512, 2048).

Each bottleneck block consisted of 1 × 11 reduction, 3 × 33 spatial, and 1 × 11 expansion convolutions with identity/projection shortcuts. After Stage 4, global average pooling fed a fully connected layer (2 units) with SoftMax.

### Implementation details

To enable like-for-like comparisons, the models shared the same input pipeline and output formulation. ReLU activations were used throughout the convolutional and dense layers (except the final SoftMax layer). Global average pooling allowed consistent transition from convolutional features to the classification head in both architectures, while dropout was applied only in the custom CNN to counteract overfitting.

Both networks were trained end-to-end using categorical cross-entropy loss for the two-class output. Their performance was monitored on a held-out validation set with early stopping and checkpoint selection based on validation loss. At inference, the class probabilities were thresholded to produce the final output, and the dataset of 1400 CBCT-derived panoramic images was randomly shuffled to prevent order bias. The data were partitioned into three subsets: 70% for training (*n* = 980), 17% for validation (*n* = 240), and 13% for testing (*n* = 180). To improve model robustness and prevent overfitting, no augmentation was applied to the training, validation, or test set predictions. The total trainable parameters for both models (∼110 thousand for the custom CNN and ∼23.5 million for ResNet-50) were calculated to highlight the complexity gap.

All computational processes were executed on a Windows 10 workstation equipped with an AMD Ryzen 9 7940HS processor, 32 GB of random-access memory, and an NVIDIA GeForce RTX 4070 Mobile graphics processing unit (8 GB of video random-access memory). The deep learning models were implemented using Python 3.9, relying on the TensorFlow framework (v2.10) with Keras. Both architectures were trained end-to-end using the Adam optimiser with the default parameters $\left(\beta_{1}=0.9,\;\beta_{2}=0.999\right)$ and an initial learning rate of 0.001. The batch size was set to 32 to fit comfortably within the graphics processing unit memory while ensuring stable gradient updates. Training was conducted for a maximum of 30 epochs, since preliminary runs indicated convergence within this timeframe. A categorical cross-entropy loss function was used for the two-class classification.

### Grad-CAM–based model interpretability

Grad-CAM was used to visualise class-discriminative regions that drove the models’ decisions.^17^ For an input radiograph and target class (anomaly or healthy), Grad-CAM computes the gradient of the class score before the SoftMax layer with respect to the feature maps of the last convolutional layer. The channel weights were obtained by global average pooling of the gradients,

(1) $$a_{k}^{c}=\frac{1}{2}\sum_{i,j}\frac{dy^{c}}{dA_{i,i}^{k}}$$

and the coarse localisation map was formed as follows,

(2) $$L_{Grad-cam}^{c}=ReLU\left(\sum_{k}a_{k}^{c}A^{k}\right)$$

which retained only features that positively influenced class CCC. For the custom CNN, this corresponded to the final 3 × 33 convolutional block (128 filters); for ResNet-50, this was taken from the last bottleneck stage (C5; 2048 channels). Heatmaps were bilinearly upsampled to 224 × 224, min to max normalised to [0,1], and overlaid on the original image using a perceptual colourmap with fixed transparency to highlight salient regions. Grad-CAM maps were generated for the predicted class for qualitative auditing and for the alternative class when inspecting failure cases, allowing assessment of whether attention was concentrated around the mental-foramen region and adjacent anatomical structures.

To ensure transparent and standardised reporting of the AI methodology, this study adhered to the checklist for AI in medical imaging^18^ guidelines; the completed checklist is provided in Table.

**Table:** Checklist for artificial intelligence in medical imaging.

Section/topic	Item #	Description
**Title/abstract**		
**Title/abstract**	1	Identification as a study of AI methodology, specifying the technology (eg, deep learning).
	2	Structured summary of study design, methods, results, and conclusions.
**Introduction**		
**Background**	3	Scientific and clinical background, including the intended use/clinical role of the AI.
	4	Study objectives and hypotheses.
**Methods**		
**Study design**	5	Prospective or retrospective study.
	6	Study goal (eg, model creation, feasibility, noninferiority).
**Data**	7	Data sources (dates, locations, settings).
	8	**Inclusion and exclusion criteria.**
	9	**Data preprocessing** (normalisation, resizing, etc.).
	10	**Selection of data subsets** (training/val/test splits).
	11	Deidentification methods.
	12	How missing data were handled.
**Ground truth**	13	Definition of ground truth reference standard.
	14	Rationale for ground truth definition.
**Model**	15	**Detailed model architecture.**
	16	Model initialisation (eg, pretraining).
**Training**	17	**Hyperparameters** (learning rate, batch size, optimiser).
	18	**Hardware and software** specifications.
**Evaluation**	19	Metrics used (accuracy, AUC, sensitivity, etc.).
**Results**		
**Data flow**	20	Flow of participants/images (total, excluded, final).
**Demographics**	21	Demographic and clinical characteristics of the dataset.
**Model performance**	22	Performance metrics estimates with confidence intervals.
	23	Estimates of test error (false positives/negatives).
**Interpretability**	24	Methods to explain model decisions (eg, heatmaps).
**Discussion**		
**Limitations**	25	**Study limitations** (bias, errors, generalisability).
**Implications**	26	Implications for practice and future research.

## Results

Across a balanced test set (anomalies and healthy cases, 95 each), ResNet-50 outperformed the compact CNN on all summary metrics. The overall accuracy improved from 71.1% (CNN) to 85.8% (ResNet-50), a +14.7-point absolute gain. The macro-averaged F1-score increased from 0.71 to 0.86. The anomaly recall – a clinically critical measure – improved from 0.68 to 0.88, reducing missed anomalies from 30 to 11 cases (a 63% reduction). With *n* = 190, the binomial 95% confidence intervals for accuracy were 0.65 to 0.78 (CNN) and 0.81 to 0.91 (ResNet-50).

**Fig. 2:**
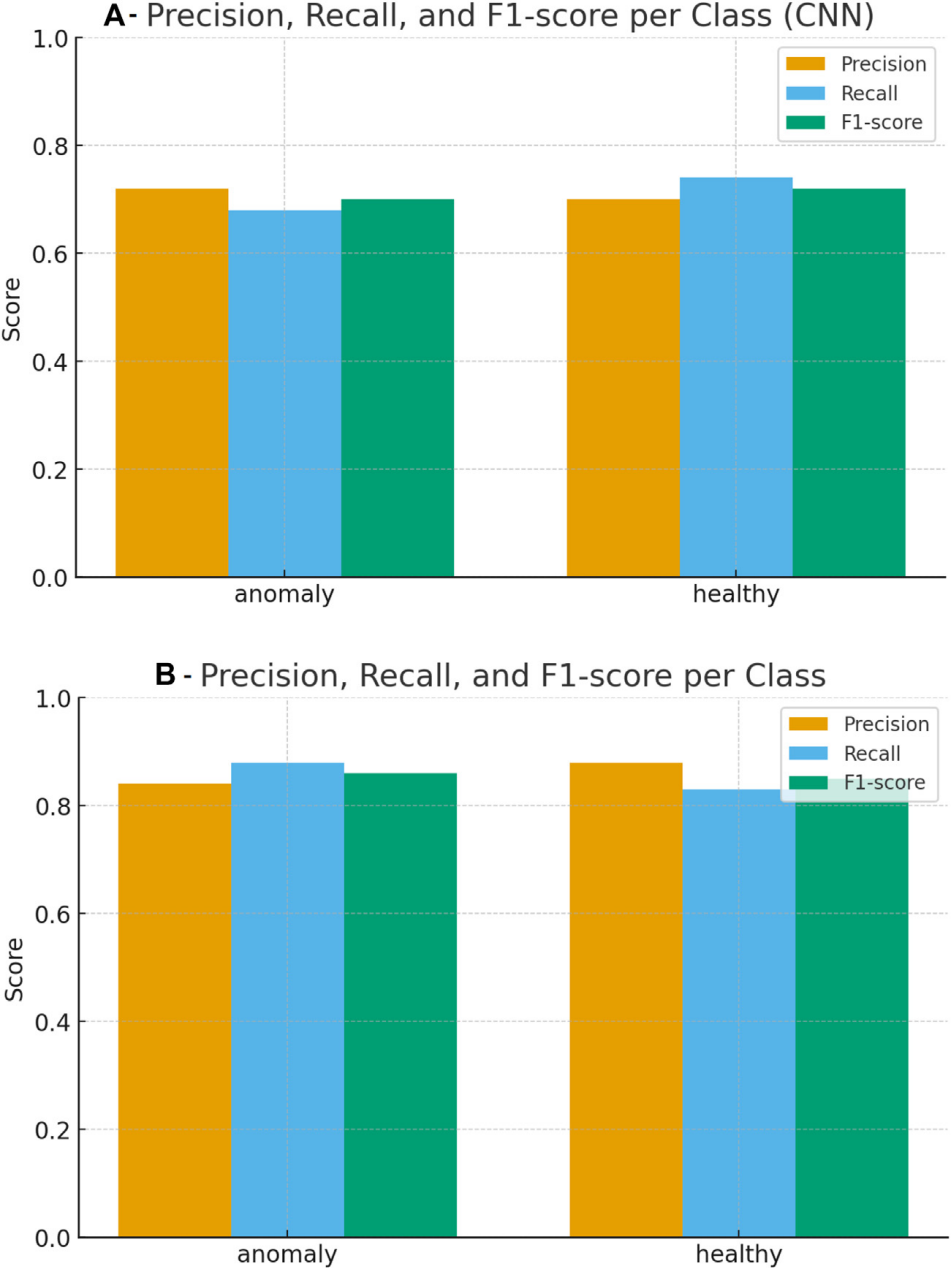
Precision, recall, and F1-score per class. (A) CNN; (B) ResNet-50.

As shown in Figure 2, the precision, recall, and F1-score of the CNN model for anomalies were 0.72, 0.68, and 0.70, respectively, while the corresponding values for healthy cases were 0.70, 0.74, and 0.72, respectively. The macro-averaged precision/recall/F1-score of the CNN model were approximately 0.71/0.71/0.71. The precision, recall, and F1-score of the ResNet-50 model for anomalies were 0.84, 0.88, and 0.86, respectively, while the corresponding values for healthy cases were 0.88, 0.83, and 0.85, respectively. The macro-averaged precision/recall/F1-score of the ResNet-50 model were approximately 0.86/0.86/0.86.

**Fig. 3:**
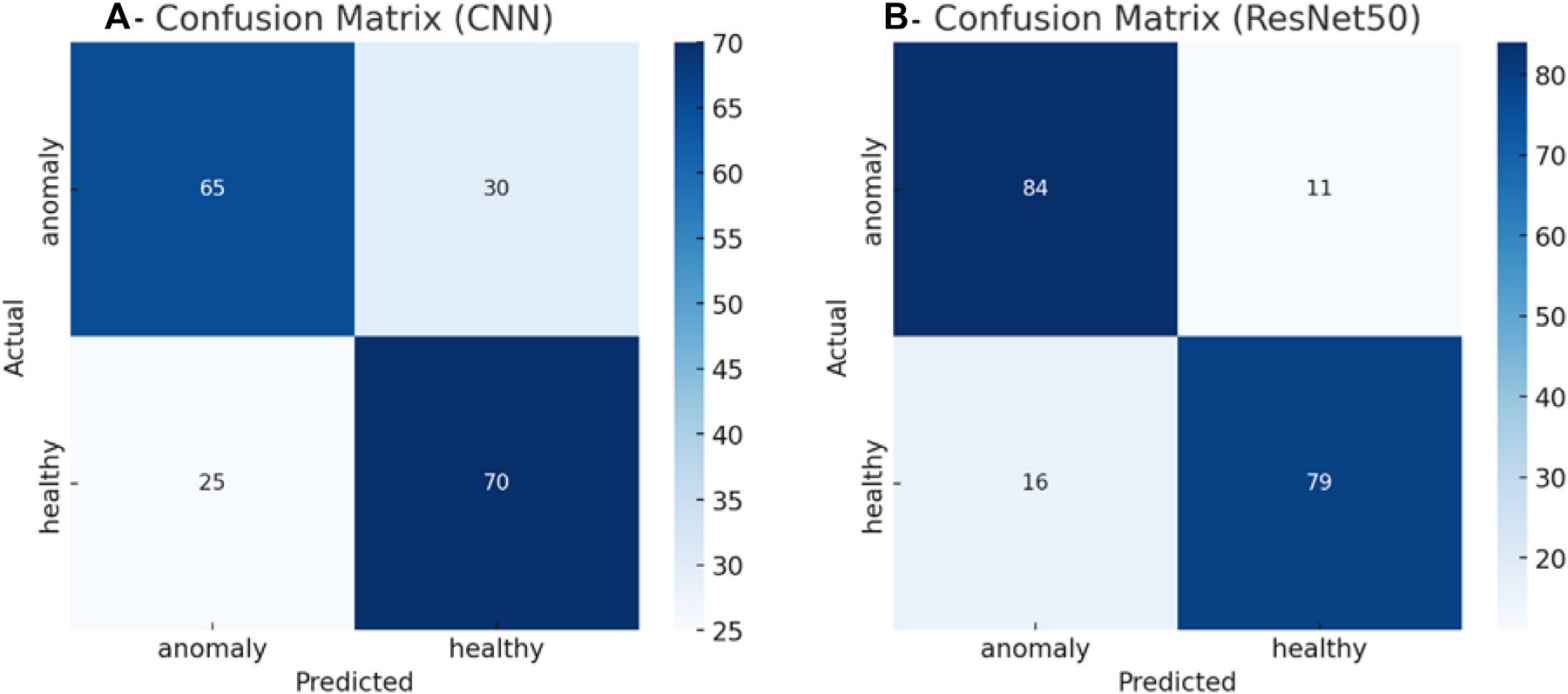
Confusion matrix of the models. (A) CNN; (B) ResNet-50.

Figure 3 presents the confusion matrices on a balanced test set (95 anomalies, 95 healthy cases). The compact CNN correctly identified 65/95 anomalies and 70/95 healthy cases (71.1% accuracy); its precision and recall for identifying anomalies were 0.72 and 0.68, respectively, and it identified 30 false-negative and 25 false-positive anomalies. In comparison, ResNet-50 identified 84/95 anomalies and 79/95 healthy cases correctly (accuracy, 85.8%); its precision and recall for identifying anomalies were 0.84 and 0.88, respectively, and it identified 11 false-negative and 16 false-positive anomalies. Thus, ResNet-50 showed a 63% reduction in missed anomalies and a notably more balanced error profile.

**Fig. 4:**
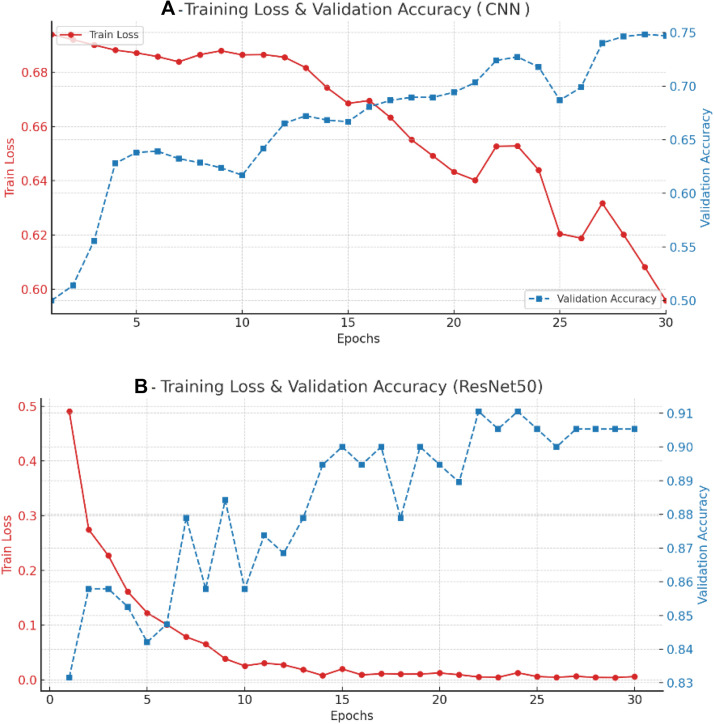
Training loss and validation accuracy of the models. (A) CNN; (B) ResNet-50.

Figure 4 compares learning dynamics for the two models: In panel A (CNN), training loss declined only modestly from ∼0.70 to ∼0.60 across 30 epochs, while validation accuracy increased slowly from 0.50 to 0.75 with noticeable fluctuations. The CNN model showed an underfitting pattern, indicating limited representational capacity relative to the task. In panel B (ResNet-50), training loss fell rapidly from ∼0.49 to ∼0.01 by ∼20 to 25 epochs, and validation accuracy increased steadily to a stable plateau around 0.90 to 0.91; the large training–validation gap reflects high capacity with mild overfitting but substantially better generalisation than the CNN.

Relative to the compact CNN, ResNet-50 provided markedly better discrimination, particularly for the anomaly class. The training curves corroborated these findings: the CNN exhibited capacity-limited learning, whereas ResNet-50 converged to a higher-accuracy plateau with substantially fewer false negatives for anomalies.

**Fig. 5:**
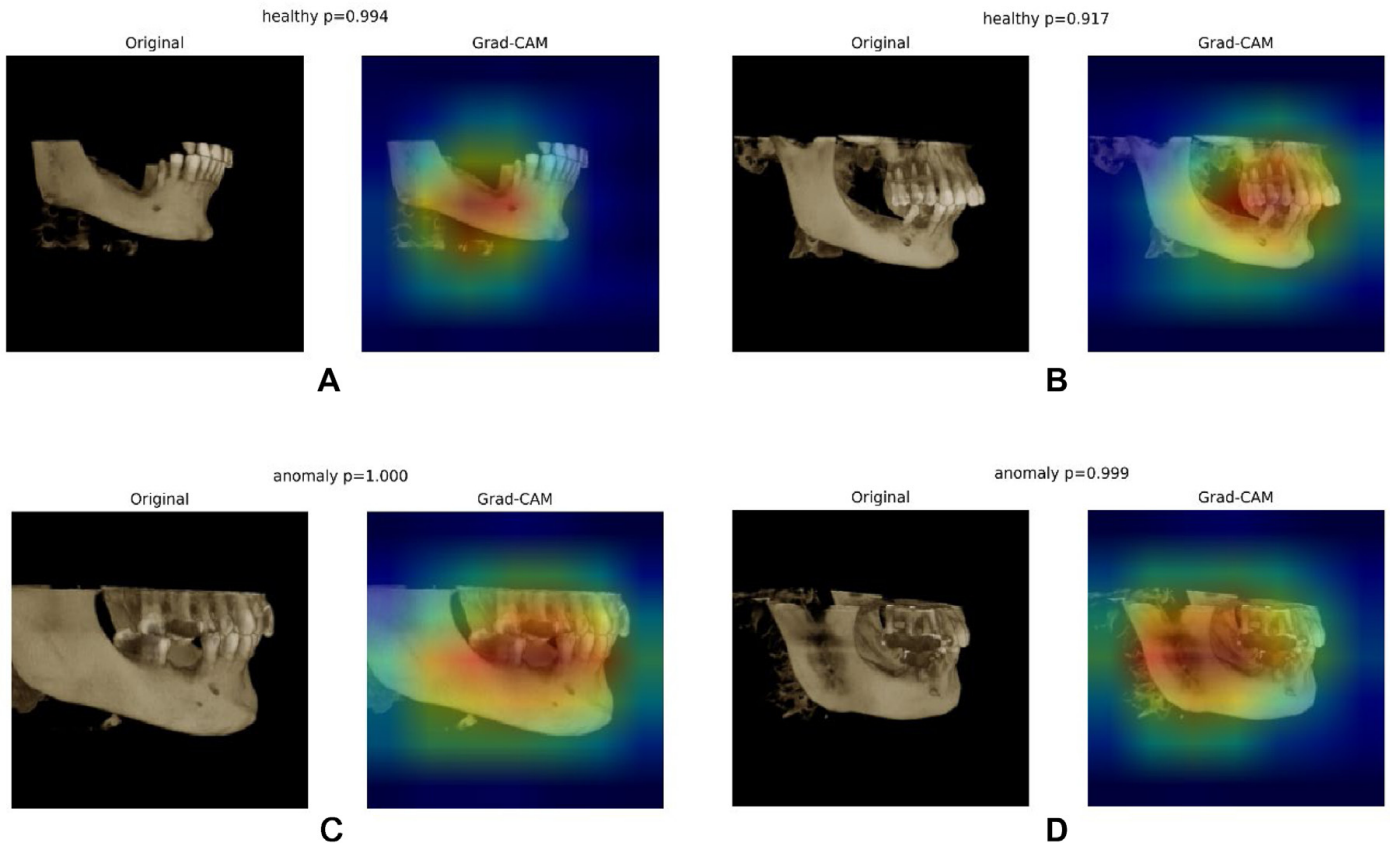
Grad-CAM visuals of the ResNet50 model. (A) Healthy-1; (B) Healthy-2; (C) Anomaly-1; (D) Anomaly-2.

Figure 5 illustrates class-discriminative attention for the representative test images. Panels A and B (both predicted healthy; *P* = .994 and *P* = .917) show compact, high-intensity Grad-CAM responses centred on the mandibular premolar region surrounding the expected location of the MF, with minimal spillover to distant structures. Panels C and D (both predicted anomaly; *P* = 1.000 and *P* = .999) display broader, asymmetric hotspots extending beyond the foramen into adjacent alveolar bone and root apices, matching the visual extent of the abnormal region. In comparison with healthy examples, anomaly maps exhibit larger, more intense activations and a shift from focal to multifocal attention, indicating that the model’s decision is driven by tissue changes proximate to the MF rather than global image artefacts. Overall, the heatmaps are localised to anatomically plausible regions for both classes, providing visual support for model reliability in this task.

## Discussion

Recognition of the MF is essential to prevent iatrogenic complications during dental and surgical procedures involving the mandible. CBCT, a radiographic imaging modality that can produce precise 3D representations of hard tissues, has become one of the most important advances in modern medical imaging.^19^

The presence of an AMF has important clinical implications, particularly for achieving adequate anaesthesia of the mental nerve and avoiding neurovascular injury during surgical interventions near the MF. The reported frequencies of AMFs vary widely across studies, ranging from 1.4% to over 20%.^20^, ^21^, ^22^ This variation may be attributable to differences in the definition and identification criteria of AMFs, imaging methodologies, and ethnic diversity. Anatomically, an AMF is defined as an additional buccal foramen with a distinct bony canal branching from the MC, and this classification excludes nutritional foramina lacking an MC connection and buccal foramina emerging from the mandibular incisive canal.^23^^,^^24^

The integration of AI techniques, particularly deep learning, into dental radiology has been accelerating recently. Deep learning models, especially CNNs, have demonstrated expert-level performance in various medical imaging domains.^25^ Given their capacity to learn from contextual information within image slices of intricate 3D anatomical structures, CNNs have demonstrated substantial promise in producing accurate findings.^26^ CNNs, which employ deep learning, have been effectively used to assess visual imagery. In a big dataset like a dataset of 2D radiograph or 3D CT scans, CNN networks may identify and separate certain patterns. CNNs can recognise a collection of pixels or voxels that constitute the interior or contour of objects of interest. By learning contextual information within image slices of complex 3D anatomical structures, CNNs can perform automatic recognition, classification, and segmentation of dental features, including anatomical landmarks.^27^ Some of the dental applications of CNNs include anatomical landmark recognition, classification, and segmentation^28^; diagnosis of dental caries,^29^ analyses of periapical lesions,^30^ cystic lesions and tumours,^31^ and maxillary sinuses^13^; and cephalometric analysis.^32^ Despite these advancements, the application of deep learning for MF and AMF detection on CBCT images remains limited.^33^ Previous studies employing U-Net architectures for MC segmentation reported moderate accuracy, with Dice similarity coefficients ranging from 0.51 to 0.63 and intersection-overunion values between 0.34 and 0.45.^34^

In this context, the novel contribution of our study was the integration of CBCT-based imaging with deep learning to automatically detect AMFs. Using a CNN-based algorithm trained on annotated CBCT datasets, the model achieved an accuracy of 85.8% in identifying AMFs, demonstrating superior performance over previously reported methods. These results highlight the potential of deep learning-assisted CBCT analysis as a reliable and efficient diagnostic tool for precise localisation of anatomical variations such as the AMF, thereby supporting clinicians in preoperative planning and risk assessment. Furthermore, the integration of AI must address ethical challenges and adhere to international standards, such as those outlined in the recent FDI Communique.^35^

Deep learning models have been used to detect and segment the MC in both CBCT scans and panoramic radiographs.^12^ However, studies describing the use of deep learning models for segmentation of the MF and mandibular foramen using panoramic radiographs and CBCT scans are limited.^36^ In our study, automatic detection of AMFs was performed using deep learning-based CNN architectures applied to 3D reformatted CBCT images, and this approach offered several distinct advantages over traditional diagnostic approaches. By leveraging CBCT’s high spatial resolution and the pattern-recognition capabilities of CNNs, our model achieved rapid, objective, and reproducible detection of anatomical variations that are often difficult to identify manually. This automation minimised observer bias, reduced diagnostic time, and enhanced consistency across large datasets. The integration of Grad-CAM visualisation further improved clinical interpretability by highlighting the regions that influence model predictions, allowing practitioners to verify anatomical relevance. In comparison with conventional manual segmentation or rule-based algorithms, our deep learning approach provided a scalable and efficient solution that can be seamlessly integrated into clinical workflows, supporting more accurate preoperative planning and reducing the risk of neurovascular complications during mandibular surgeries.

The primary limitation of this study was the use of 2D panoramic reconstructions rather than full volumetric CBCT data as the input for the AI models. While the segment images were established using detailed cross-sectional analysis (axial, sagittal, and coronal), the AI models were restricted to 2D features. Consequently, subtle anatomical variations or AMFs obscured by superimposition in the panoramic projection may be less detectable by the model than by a human observer viewing the full 3D volume. Future studies utilising 3D-CNNs on voxel data could potentially yield higher sensitivity for these complex anatomical structures.

## Conclusions

Our findings demonstrated that deep learning-based analysis of CBCT images can effectively and accurately detect AMFs, providing a valuable tool for maxillofacial diagnostics and surgical planning. The ResNet-50 architecture outperformed the custom CNN, achieving higher accuracy and reliability while maintaining clinically interpretable results through Grad-CAM visualisation. These findings highlight the potential of AI to enhance diagnostic precision, reduce human error, and streamline radiological workflows. Future studies with larger and more diverse datasets may further improve model generalisation and support the integration of AI-assisted systems into routine dental and surgical practice.

## Ethics statement and consent to participate

Ethical approval for this study was obtained from the Non-Interventional Ethics Committee of Cankiri Karatekin University (Approval Code: 9e480299f39d4d9b; Approval Date: 4 December 2024). All procedures performed in this study involving human participants were conducted in accordance with the ethical standards of the institutional and/or national research committee and with the 1964 Helsinki Declaration and its later amendments. Informed consent to participate in this study was obtained from all individual participants included in the research. A copy of the participant consent form is available upon request.

## Consent for publication

Consent for publication was obtained from all individual participants whose data or images were included in this study.

## Author contributions

Zuhal Ovuz conceptualised and supervised the study and contributed to manuscript preparation. Eda Gursu Sahin and Zuhal Ovuz performed the CBCT image labelling and data preparation. Taha Etem developed and conducted the artificial intelligence analysis using convolutional neural networks. All authors reviewed and approved the final version of the manuscript.

## Conflict of interest

The authors declare that they have no known competing financial interests or personal relationships that could have appeared to influence the work reported in this article.

## Appendix Supplementary materials

Image, application 1
