# Supplementary material for: Automated Identification of Accessory Mental Foramen Using Cone-Beam Computed Tomography and Convolutional Neural Networks
Source: Int Dent J. 2026 Feb 24;76(2):109428. doi: 10.1016/j.identj.2026.109428 (PMC13080463; doi:10.1016/j.identj.2026.109428)
Supplement: Supplementary file 1 [file mmc1.pdf]

Certificate of Elsevier Language Editing Services

The following article was edited by Elsevier Language Editing Services:

AUTOMATED IDENTIFICATION OF ACCESSORY MENTAL FORAMEN USING CONE BEAM COMPUTED TOMOGRAPHY AND CONVOLUTIONAL NEURAL NETWORKS

Ordered by:

zuhal ovuz

Estimated Delivery date:

2025-12-30

Order reference:

ASLESTD1122005
